# Supplementary material for: Neuromuscular Control before and after Independent Walking Onset in Children with Cerebral Palsy
Source: Sensors (Basel). 2021 Apr 12;21(8):2714. doi: 10.3390/s21082714 (PMC8069021; doi:10.3390/s21082714)
Supplement: Supplementary file 1 [file sensors-21-02714-s001.pdf]

Supplemental material

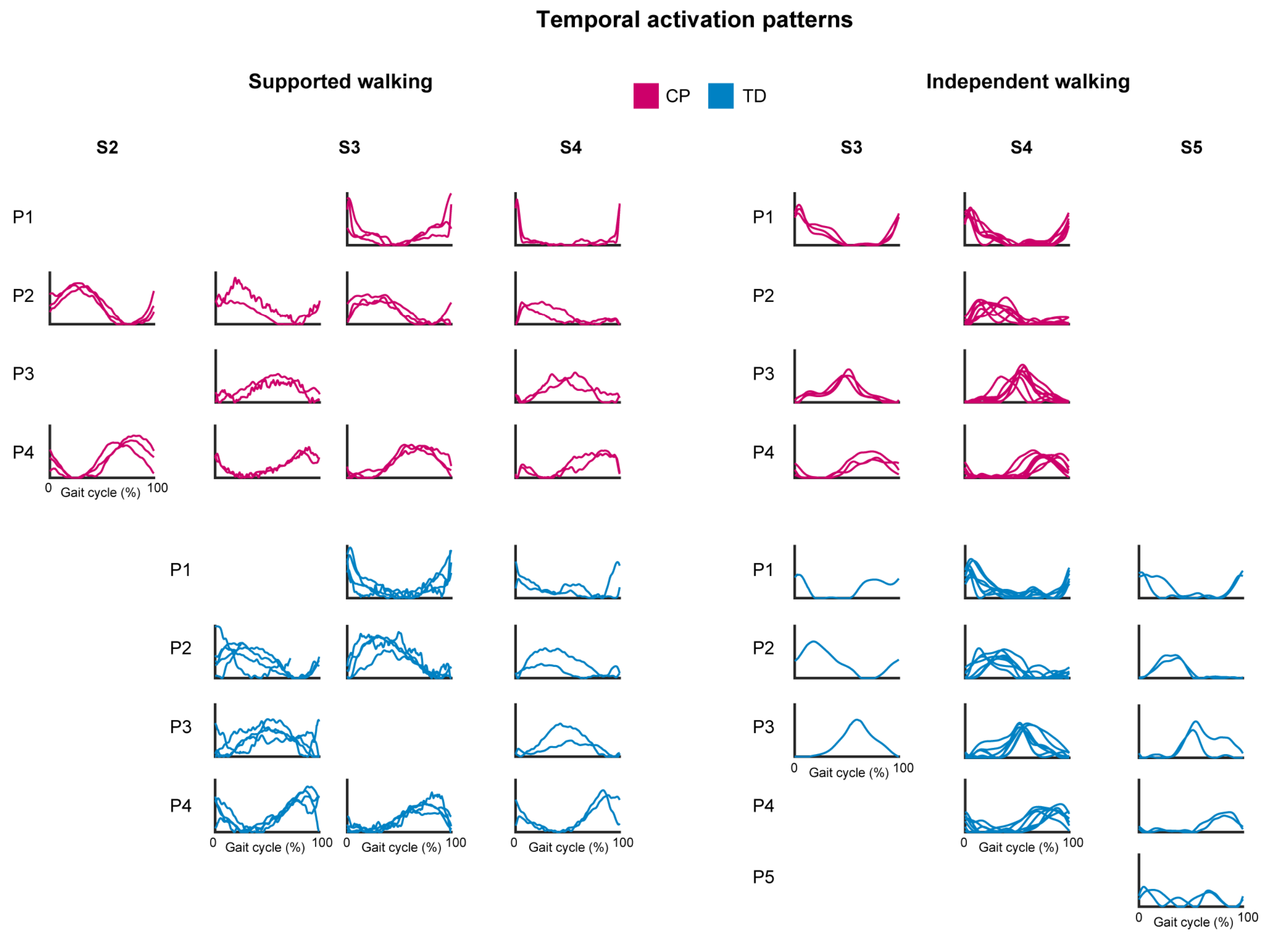

Figure S1. Individual temporal activation patterns. Bilateral EMG analysis: temporal activation patterns (P1 to P5) for the number of synergies (S2 to S5) defined for each participant (separate lines) based on the variability accounted for (VAF) threshold of 85% for the cerebral palsy (CP) and typically developing (TD) group in the supported walking group (*left*) and independent walking group (*right*).
